# Why some do but too many don’t? Barriers and enablers to physical activity in regional Tasmania – an exploratory, mixed-methods study

**DOI:** 10.1186/s12889-022-13001-6

**Published:** 2022-03-31

**Authors:** Sisitha Jayasinghe, Robert Soward, Timothy P. Holloway, Kira A. E. Patterson, Kiran D. K. Ahuja, Roger Hughes, Nuala M. Byrne, Andrew P. Hills

**Affiliations:** 1 College of Health and Medicine, University of Tasmania, Hobart, Tasmania Australia; 2 College of Arts, Law and Education, University of Tasmania, Hobart, Tasmania Australia

**Keywords:** Obesity, Physical activity, Environment, Infrastructure, Regional

## Abstract

**Background:**

The interconnectedness of physical inactivity and sedentarism, obesity, non-communicable disease (NCD) prevalence, and socio-economic costs, are well known. There is also strong research evidence regarding the mutuality between well-being outcomes and the neighbourhood environment. However, much of this evidence relates to urban contexts and there is a paucity of evidence in relation to regional communities. A better understanding of available physical activity (PA) infrastructure, its usage, and community perceptions regarding neighbourhood surroundings, could be very important in determining requirements for health improvement in regional communities. The aims of this research were to 1. Explore and evaluate the public’s perception of the PA environment; and 2. Evaluate the quantity, variety, and quality of existing PA infrastructure in regional Northwest (NW) Tasmania.

**Methods:**

A mixed methods approach guided data collection, analysis, and presentation. Quality of PA infrastructure was assessed using the Physical Activity Resource Assessment (PARA) instrument and public perception about PA environment was evaluated using the International Physical Activity Questionnaire – Environmental (IPAQ-E) module. Quantitative data were analysed using descriptive summative methods and a team-based researcher triangulation approach was utilised for qualitative data.

**Results:**

Overall, a wide array of high-quality PA infrastructure (with minimal incivilities such as auditory annoyance, litter, graffiti, dog refuse, and vandalism etc.) was available. Survey respondents rated neighbourhoods positively. The overall quality of PA infrastructure, rated on a scale from 0 to 3, was assessed as high (all rated between 2 to 3) with minimal incivilities (rated between 0 and 1.5). Of note, survey respondents confirmed the availability of numerous free-to-access recreational tracks and natural amenities across the 3 local government areas (LGAs) studied. Importantly, most respondents reported minimal disruption to their routine PA practices due to the *COVID-19* pandemic.

**Conclusion:**

This exploratory research confirmed the availability of a wide range of high-quality PA infrastructure across all three LGAs and there was an overwhelming public appreciation of this infrastructure. The challenge remains to implement place-based PA interventions that address extant barriers and further increase public awareness and utilisation of high-quality PA infrastructure.

**Supplementary Information:**

The online version contains supplementary material available at 10.1186/s12889-022-13001-6.

## Background

The interconnectedness of physical inactivity and sedentarism, obesity, non-communicable disease (NCD) prevalence, and socio-economic costs, are well documented [1, 2]. A range of approaches have been posited to thwart the inactivity pandemic, including the identification and management of ‘individual determinants.’ Considerable attention has also been paid to the ‘person-environment fit model’ [3] representative of the mutuality between well-being outcomes and the neighbourhood environment.

Empirical evidence has highlighted numerous features of neighbourhoods such as cost of access, safety supports, amenities, traffic conditions, transit access, aesthetics etc. and their potential impact on physical activity (PA) patterns [4, 5]. Neighbourhoods conducive to PA can directly influence communal activity and social engagement patterns [6, 7]. In urban contexts, access to PA facilities, convenient and proximate access to destinations, high residential density, land use, perceived safety and availability of exercise equipment, have all been cited as potential correlates (largely base on cross-sectional associations) [8]. In contrast, there is a paucity of evidence in regional communities [9–11]. The phrase, ‘rural and remote’, encompasses all regional areas outside Australia’s major cities, using the Australian Standard Geographical Classification System. Research suggests that compared with those living in urban settings, rural residents are likely to experience different challenges to maintain an active lifestyle [8, 9, 12]. Less than 50% of the Australian adult population meets the current recommendations for PA [13] and the prevalence of inactivity is higher amongst individuals living in rural settings, mainly due to lack of, or perceived lack of PA opportunities and poor functionality of available infrastructure [14, 15].

In addition, evidence, including in Australia, is replete with reports of negative associations between socio-economic status (SES) and population-wide PA levels (particularly leisure-time, and transport-related PA) [16, 17]. For example, Giles-Corti et al. indicated that people living in low-SES neighbourhoods were less likely to use recreational facilities and engage in adequate levels of PA [18]. Similar findings have been reported in other developed economies, including in the United States [19]. One of the reasons cited for lower engagement in PA in low-SES areas is lack of accessibility to infrastructure [18–20]. Rural and regional Tasmania, including the Northwest (NW), is characterised by areas of low-SES and has some of the poorest PA participation rates in the country [21, 22]. Environmental factors may also act as barriers or enablers to PA participation and whilst not exclusive to regional communities [23], could be a key factor regarding engagement in sufficient amounts of PA.

The availability of pertinent infrastructure, and perception of neighbourhood surroundings (e.g., quality and accessibility), can also impact participation in PA [24–26]. However, there is considerable conjecture pertaining to environmental perceptions and activity patterns [27]. As such, a better understanding of PA infrastructure availability, its usage, and community perceptions regarding neighbourhood characteristics could be very important to better understand how to improve the health status of residents in regional communities.]

Therefore, the aims of this research were to:

1. Explore and evaluate the public’s perception of the PA environment in rural/remote/regional communities.
2. Explore and evaluate the quantity, variety, and quality of existing PA infrastructure in NW Tasmania.

## Methods

This study was part of a larger obesity prevention effort, the Critical Age Periods Impacting the Trajectory of Obesogenic Lifestyles (CAPITOL) Project, undertaken by the University of Tasmania in NW Tasmania across three regional Local Government Areas (LGAs), Burnie, Devonport, and Circular Head. Regional Australia includes all the towns, small cities and areas that lie beyond the major capital cities (Sydney, Melbourne, Brisbane, Perth, Adelaide, and Canberra) [28]. A LGA is an administrative division with responsibility vested in local government (Local Government Act 1993). Briefly, the selected LGAs are classified as Remoteness Area 2 (Inner Regional Australia) and 3 (Outer Regional Australia), according to the Australian Statistical Geography Standard classification system.

### Study participants

Community members over 18 years of age residing in the Burnie, Devonport, or Circular Head LGAs were invited to participate in the research. Invitations were extended through a variety of web (e.g., University and local council web and social media pages) and print media (e.g., local newspapers), and surveys were made available in both print and online forms to optimize outreach. All procedures were approved by the Human Research Ethics Committee (Tasmania) Network; H0016117 and conformed to the guidelines of the National Health and Medical Research Council’s National Statement on Ethical Conduct in Human Research 2007 (Updated 2018). Submission/ return of completed surveys was contingent upon the provision of consent from all individuals.

### Variety of physical activity infrastructure

**Fig. 1:**
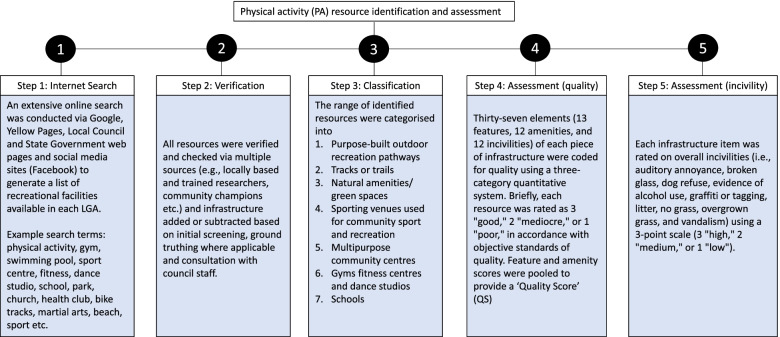
Physical activity infrastructure identification and assessment process

A comprehensive list of the PA infrastructure across each LGA was generated by research staff from online search engines (e.g., Google, Bing), social media (Facebook), Yellow Pages and relevant LGA and State Government webpages (Fig. 1). This list was verified and checked via multiple sources including trained researchers and community champions. Infrastructure was added or subtracted based on initial screening, ground truthing where applicable, and consultation with Council staff. For the purposes of this research, only facilities and spaces with public access (not private homes and worksites), were considered as PA infrastructure. The infrastructure was then categorised into purpose-built outdoor recreation pathways, tracks or trails, natural amenities or green spaces, sporting venues used for community sport and recreation, multipurpose community centres, gyms, fitness centres and dance studios, and schools, based on previously published approaches [20, 29].

### Quality of physical activity infrastructure

Quality of infrastructure was assessed using the Physical Activity Resource Assessment (PARA) instrument [30]. A four-person research team underwent training and pilot tested the PARA instrument prior to being deployed in the field, and all assessments were undertaken from June to July 2020. To minimise subjective bias, at least two persons independently assessed each infrastructure item.

### Perceptions of the physical activity environment

Public perception of the PA environment was evaluated using the International Physical Activity Questionnaire – Environmental module (IPAQ-E) [31]. The IPAQ-E is a 17-item questionnaire (7 core and 10 optional items) that measures attributes of the built neighbourhood, and social environments hypothesized, or known, to be related to PA. These include, perceived safety of local surroundings, attractiveness, pleasantness of walking near home, proximity of shops, availability of walking and biking routes, and traffic patterns. Three additional open-ended questions were included to elicit further information about 1. Barriers to regular activity, 2. Impact of *COVID-19* on activity patterns, and 3. Other location-specific challenges to being active (Table 1).

**Table 1:** Structure of IPAQ-E and open-ended questions

Theme	Question	Scoring system
Proximity	Many shops, stores, markets, or other places to buy things I need are within easy walking distance of my home. Would you say that you...	Four-point Likert response scales ranging from strongly disagree to strongly agree, as well as don’t know or doesn’t apply response options, were used for all variables.
	It is within a 10–15-min walk to a transit stops (such as bus stop) from my home. Would you say that you...	
	There are many places to go within easy walking distance of my home. Would you say that you...	
Availability/ variety	There are footpaths on most of the streets in my neighbourhood. Would you say that you...	
	There are facilities to bicycle in or near my neighbourhood, such as special lanes, separate paths or trails, shared use paths for cycles and pedestrians. Would you say that you...	
	My neighbourhood has several free or low-cost recreation facilities, such as parks, walking trails, bike paths, recreation centres, playgrounds, public swimming pools, etc. Would you say that you...	
Safety	The crime rate in my neighbourhood makes it unsafe to go on walks during the day. Would you say that you...	
	The crime rate in my neighbourhood makes it unsafe to go on walks at night. Would you say that you...	
Traffic	There is so much traffic on the streets that it makes it difficult or unpleasant to walk in my neighbourhood. Would you say that you...	
	There is so much traffic on the streets that it makes it difficult or unpleasant to ride a bicycle in my neighbourhood. Would you say that you...	
	There are many four-way intersections in my neighbourhood. Would you say that you...	
Peer influence	I see many people being physically active in my neighbourhood doing things like walking, jogging, cycling, or playing sports and active games. Would you say that you...	
Aesthetics/ maintenance	There are many interesting things to look at while walking in my neighbourhood. Would you say you...	
	The footpaths in my neighbourhood are well maintained (paved, with few cracks) and not obstructed. Would you say that you…	
	Places for bicycling (such as bike paths) in and around my neighbourhood are well maintained and not obstructed. Would you say that you...	
In addition to the above, participants were requested to answer 3 open-ended questions (1. Are there any barriers towards you being physically active? 2. How has COVID-19 and government restrictions affected your physical activity? 3. Is there anything you wish to add that you think is relevant?)		

Participants were initially recruited via online invitations extended through LGA websites, their social media pages, local newspapers, and the University of Tasmania Facebook page. The online recruitment drive was supplemented with a targeted letterbox drop of paper surveys to the most populous suburbs in each LGA.

### Quantitative data analysis

Thirty-seven elements (13 features, 12 amenities, and 12 incivilities) of each piece of infrastructure were coded for quality using a three-category quantitative system. Briefly, each resource was rated as 3 “good,” 2 “mediocre,” or 1 “poor,” in accordance with objective standards of quality. Additionally, each infrastructure item was rated on overall incivilities (i.e., auditory annoyance, broken glass, dog refuse, evidence of alcohol use, graffiti or tagging, litter, no grass, overgrown grass, and vandalism) using a 3-point scale (3 “high,” 2 “medium,” or 1 “low”) to derive an Incivility Score (IS) [29]. Feature and amenity scores were pooled to provide a ‘Quality Score’ (QS), with 3 being the highest quality. The QS was derived by calculating the mean of (rated) feature and amenity items for each infrastructure asset. The overall IS was derived by calculating the mean of (rated) incivility items for each infrastructure, again using the 3-point scale with 3 being the highest level of incivility.

### Qualitative data analysis

Responses to the 17 IPAQ-E questions regarding the local physical environment (defined as a 10- to 15-min walk from the home) were collated and presented as percentages for each item using a four-point Likert scale. A team-based, researcher triangulation approach to qualitative thematic data analysis was used to manage and interpret answers to the open-ended questions [32]. Briefly, researchers read and re-read all answers, identified main themes, and refined them prior to generating the ‘summary table’. All transcripts of open-ended questions were deconstructed and distributed to analytical teams of at least 2 researchers (authors on this paper). Each researcher thematically analysed data independently, through familiarisation; searching for themes (meaning); refining themes; determining the story of each of them and documenting. Subsequently, the primary author, who analysed data across all questions, coordinated and arbitrated triangulation discussions with each analytical pair. Agreement between all members regarding the themes was attained through iterative, inductive, and reflexive means to maintain rigour [33, 34].

## Results

Descriptive characteristics of the survey cohort are provided in Table 2. A total of 344 adults, with a mean age of 53 years, responded to the survey with nearly two-thirds being female. Most respondents lived in ‘detached single family homes’ and in most instances, owned 1 or 2 motor vehicles (Table 2). A wide variety of PA infrastructure is available to NW Tasmanian residents with ~ 30% of facilities incurring no usage costs (Recreation tracks and natural amenities; Table 2).

**Table 2:** Descriptive characteristics of survey respondents and LGAs

	Burnie (*n* = 101)	Devonport (*n* = 181)	Circular Head (*n* = 49)	Total
Survey respondents^c^				
Gender				
Male	33	57	11	101
Female	66	119	35	220
Did not disclose gender	0	3	1	4
Mean age	59	53	57	
Education				
Grade ≥ 10	92	172	46	310
Housing				
Detached single family housing	78	134	41	253
Apartments 4–12 stories	17	27	3	47
Single family residences and townhouse	5	13	5	23
Rural living zone	1	7	0	8
Vehicle ownership				
No motor vehicles	4	10	2	16
1 motor vehicle	33	51	13	97
2 motor vehicles	37	72	19	128
3 or more motor vehicles	27	44	15	86
Don’t know/ unsure	0	2	2	2
All LGAs				
Variety of PA infrastructure				
Recreation tracks	3	12	3	18
Natural amenities/ green spaces	12	9	18	39
Sporting venues	14	16	8	38
Multipurpose community Centre	2	3	3	8
Gymnasia	13	19	2	34
School	15	14	8	37
Demographics				
Population (> 18 years)^a^*	18,919	14,308	5917	
Geographical area (km^2^)^a^	111	611	4898	
Health status				
Prevalence of overweight and obesity (% of adults > 18 years)^b^	76	59	70	
Prevalence of insufficient moderate/vigorous PA (18–64 years)^b^	16	18	26	
Prevalence of insufficient muscle strengthening (18–64 years)^b^	77	75	78	

^a^Source: Australian Bureau of Statistics 2016 Census, IRSAD: Index of Relative Socio-economic Advantage and Disadvantage (SEIFA figures reflect higher advantage with a higher score)

^b^Report on the Tasmanian Population Health Survey 2019

^c^Thirteen respondents did not indicate their ‘LGA’ and were not included in the final calculations

### Quality of PA infrastructure

**Fig. 2:**
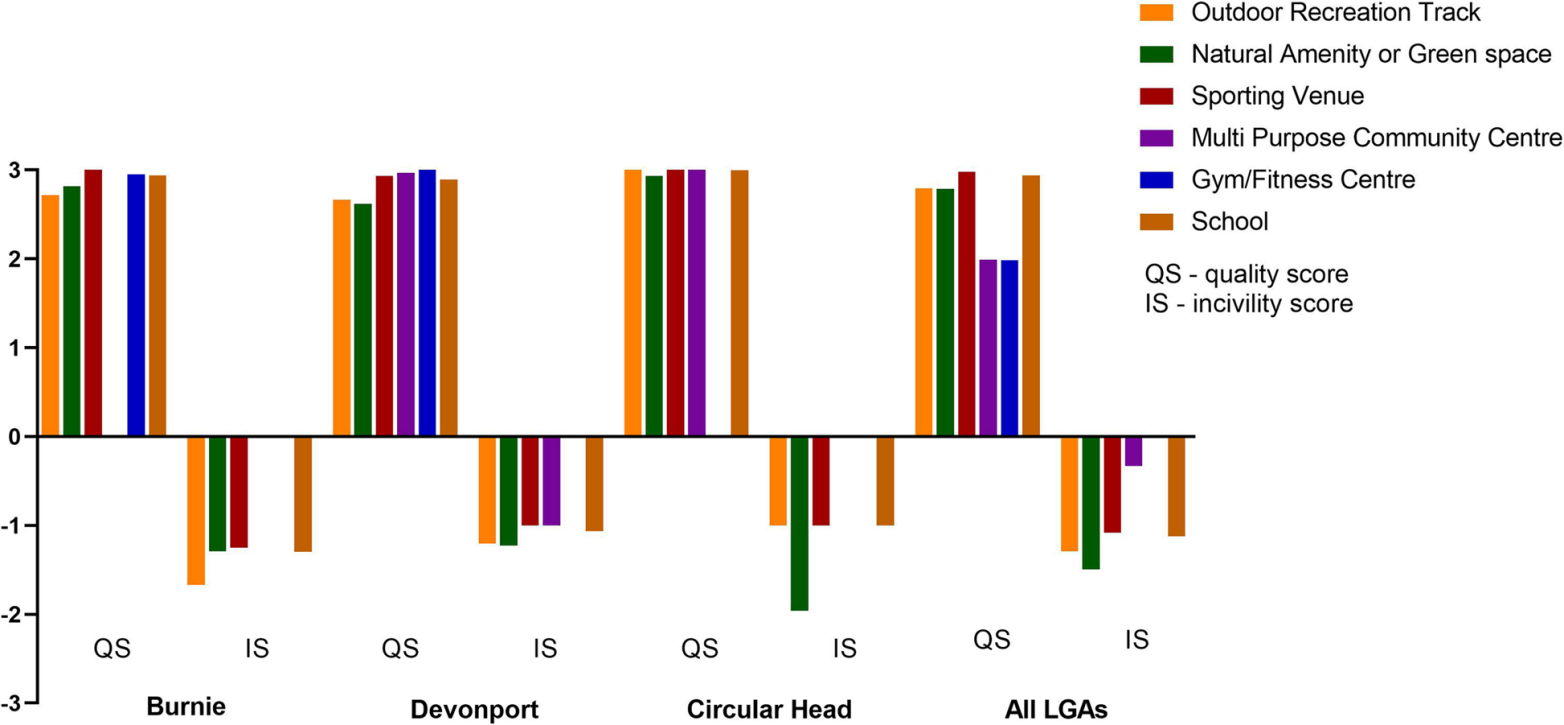
Quality of available physical activity infrastructure

**Fig. 3:**
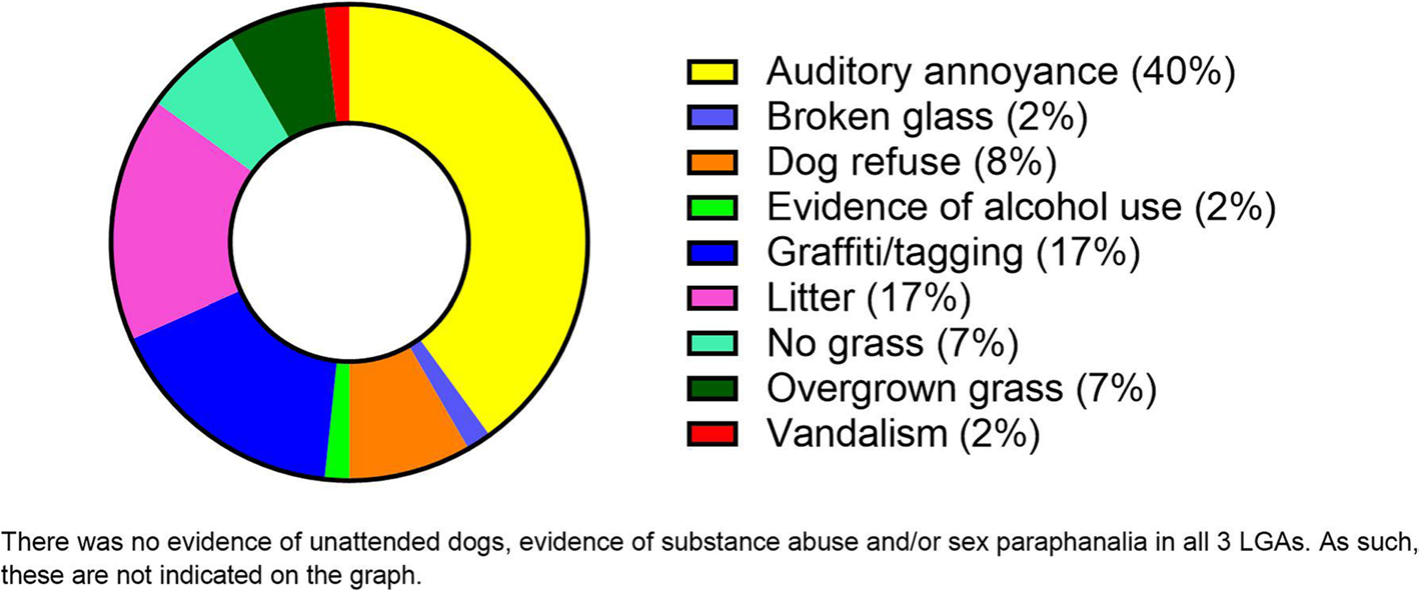
Types (% of total) of incivilities observed

**Fig. 4:**
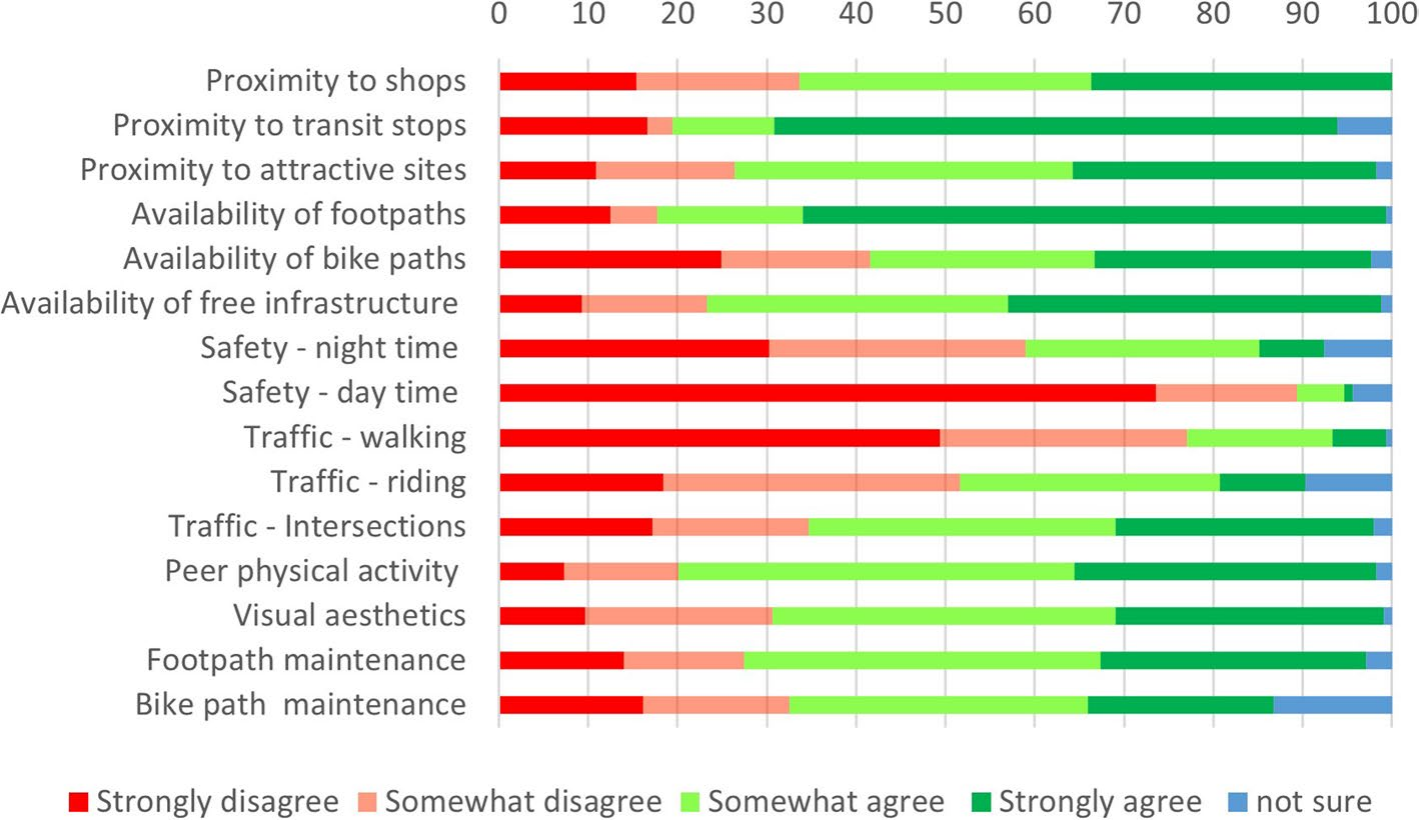
Public perception of physical activity environment in all 3 LGAs

Despite subtle differences, the overall quality of available PA infrastructure was generally high (range 2–3; Fig. 2). In contrast, incivilities were low (range 0–1.5) with auditory annoyance, graffiti or tagging and litter being the most frequently recorded incivility type (Fig. 3). Overall perceptions of the PA settings across the three LGAs in NW Tasmania were positive (Fig. 4).

### Proximity

Two-thirds of survey respondents indicated they had easy access to shops and amenities and approximately 75% confirmed convenient proximity to other important services such as transit stops (Fig. 4).

### Availability and safety

Over 80% of the respondents indicated adequate footpaths and more than 75% indicated adequate availability of free-to-access PA options. In contrast, some 40% of respondents indicated poor availability of bike paths. More than 75% of respondents had no safety concerns during daylight hours, but some (~ 33%) were more apprehensive at night (Fig. 4).

### Traffic

Many (77%) did not view ‘traffic conditions while walking’ as an impediment to being active, in contrast to 52% of respondents who perceived traffic as an impediment while cycling (Fig. 4).

### Visual aesthetics and maintenance

Almost 70% of respondents were satisfied with the maintenance and upkeep of their neighbourhoods with positive perceptions about the visual and aesthetic appeal.

### Infrastructure preference and barriers to participation

**Fig. 5:**
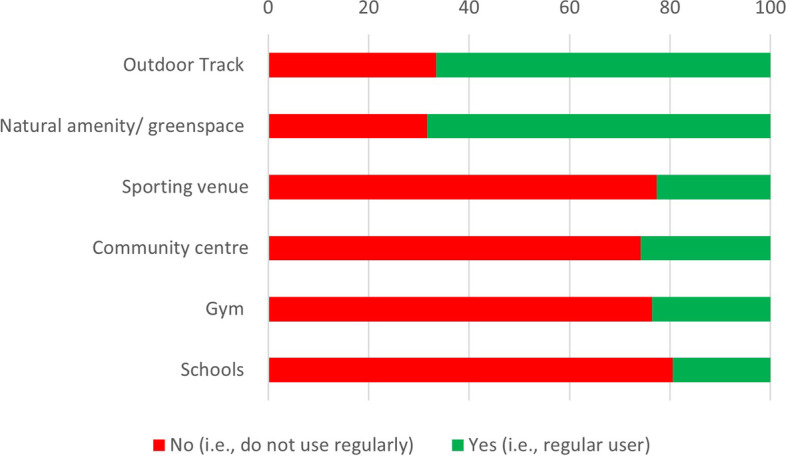
Public preference for different infrastructure types

Close to 70% of participants indicated outdoor recreation tracks and natural amenities or green space as their infrastructure of choice for regular PA (Fig. 5). Further, in response to open-ended survey questions, approximately 50% of respondents indicated there were no significant barriers to being physically active and that the *COVID-19* pandemic did not have a significant impact on their PA habits (Table 3).

**Table 3:** Barriers/challenges (% of total responses) to regular physical activity in NW Tasmania

	Burnie (*n* = 101)	Devonport (*n* = 181)	Circular Head (*n* = 49)
***Are there any barriers towards you being physically active?***			
Insufficient time	4	2	4
Physical impairments	16	16	6
Lack of infrastructure	7	4	4
Negative perceptions around safety	3	3	4
Inclement weather	3	2	4
Lack of motivation	4	3	11
No significant barrier	46	48	47
Other/ no response	16	22	19
***How has COVID-19 and government restrictions affected your physical activity?***			
No effect at all (i.e., alternate modes for PA)	59	49	60
Predominantly negative (e.g., isolation loneliness)	14	13	15
Predominantly positive (i.e., increased PA due to additional time availability)	6	6	0
Temporary inconvenience due to suspension of facility access	6	9	10
Other/ no response	15	23	15
***Is there anything you wish to add that you think is relevant?***			
Council assistance in identification of facilities and programs	4	4	2
Bike track/ pathway infrastructure improvements	7	3	9
Improvements to existing infrastructure and addition of new facilities	5	9	6
Other/ no response	84	84	83

## Discussion

This exploratory research evaluated the quantity, variety, and quality of PA infrastructure in three LGAs with a concomitant evaluation of public perception of the neighbourhood PA environment. Overall, an array of high-quality PA infrastructure (with minimal incivilities) was available, and survey respondents rated neighbourhoods positively. The overall quality of PA infrastructure was assessed as high (all rated between 2 to 3) with minimal incivilities (rated between 0 and 1.5). This investigation confirmed the availability of numerous free-to-access recreational tracks and natural amenities across the 3 LGAs, and importantly, most respondents reported minimal disruption to their routine PA related to the *COVID-19* pandemic.

The quality and usability of PA infrastructure, visual appeal, and perceptions of safety are all important elements [35]. Evidence indicates that people tend to be more active in neighbourhoods that are visually appealing with lower numbers of incivilities [36]. On the flipside, incivilities such as litter, graffiti, dog mess and vandalism can deter people from being physically active and collectively, contribute to negative perceptions about safety [35, 37, 38]. Lower incivility numbers may also impact PA involvement and consequential advantages including reductions in the prevalence of overweight and obesity [37]. Based on our observations, litter, auditory annoyances, dog refuse and graffiti were the most common incivilities. Although minimal in total, many of the incivilities reported were as a consequence of the geographical proximity of assets to industrial precincts or major roads. Overall, QS outweighed IS for 100% of PA infrastructure assessed reinforcing the level of attractiveness and visual appeal of the region. One would posit that this appeal should assist residents to be sufficiently motivated to engage in PA.

Greater anxiety about crime (perceived or real), and concerns regarding personal safety are important considerations in the PA participation patterns of many adults [35, 39, 40]. For example, adequate lighting (or lack thereof) can be a key component of perception of safety with the potential to impact PA behaviour [20]. In predominantly urban environments, presence of lighting has repeatedly been highlighted as the most important environmental feature that affects the perception of safety [41, 42].

Affordability of quality PA infrastructure and SES are intertwined, with previous research indicating that poorer neighbourhoods are usually the most affected [20, 43, 44]. One might contend that a regional area such as NW Tasmania may be particularly challenged in this context as socio-economic disparities are widespread, and the prevalence of several inactivity-related chronic conditions (including overweight and obesity), is high [22]. Our observations contradicted this notion with a total of 47 free-to-access recreation tracks and natural amenities with minimal incivilities available across the three LGAs. This is despite the SES level in the NW being lower than the country-wide average [45]. Our objective observations were further confirmed by survey respondents’ acknowledgement of the conduciveness of their ‘living environments’ to active recreation. These findings suggest that NW Tasmanian residents have ample opportunity (i.e., infrastructure) to be habitually physically active, in spite of socio-economic challenges. What might be done to assist these well-intentioned communities be more active?

One of the potential opportunities relates to the use of ‘social enterprises’ in each LGA, such as community sporting organisations, to progress local development agendas [46]. This is a potentially potent opportunity given that traditionally, engagement in community sport is an important element of the social fabric in NW Tasmania. The very low reported usage of (~ 20% of total responses) ‘sporting facilities’ is intriguing given the traditional interest in sport and activity (Fig. 4). Life-long health benefits of participation in sport are well documented [47] although engagement in sporting pursuits can depend on a myriad of factors, including gender, SES and geographical location [48, 49]. Australian data indicate a positive correlation between sport and PA participation and SES, and a negative association with remoteness and rurality [50]. Accordingly, initiatives to optimize the utilisation of community sporting facilities should be prioritised in this region.

Contrary to our expectations, most survey respondents reported uninhibited PA routines during the *COVID-19* pandemic. It is difficult to predict the longer-term effects of the pandemic, however, significant concerns have been flagged regarding the effects of *COVID-19* on existing levels of physical inactivity [51]. For instance, some of the pandemic mitigation measures (lockdowns, shelter in place, social distancing etc.) may impact accessibility to PA infrastructure, and therefore, levels of PA. The abundance of walkable areas, natural amenities, and green spaces in NW Tasmania may have enabled local residents to be relatively uninterrupted in recreation opportunities and is consistent with respondents’ perception of minimal disruption to routine habits during the pandemic (Table 2). However, some respondents were negatively impacted by social isolation due to *COVID-19* restrictions (Table 2). As existing evidence highlights a strong association between isolation and lack of social interaction and inactivity across a wide age range, [52–54] concerted efforts are required to assist in the maintenance or improvement of the mental health of communities in the NW.

There are also numerous examples of associations between insufficient motivation and time with reductions in leisure-time PA [55, 56] and in particular, lack of social support [57]. The majority of survey respondents reported seeing ‘many people being physically active in their neighbourhood’, and ‘there being many interesting things to look at while walking in their neighbourhoods’. In summary, it appears there is adequate social and environmental supports for NW Tasmanian adults to be sufficiently physically active. It is noteworthy that a small number of residents perceived poor health and lack of infrastructure as barriers to PA. Both perceptions warrant further attention in larger scale future research. The use of validated (both construct and content) assessment tools is a significant strength of this study [20, 30, 58]. The use of open-ended questions to bookend the survey is also noteworthy and likely improved data quality as previously referenced [59]. Nevertheless, the generalisability of the findings to other regions may be limited due to the relatively low number of survey responses and potential lack of representativeness of the wider community. Conceptually, sample adequacy in qualitative research is an ongoing debate with epistemological, methodological, contextual, and practical necessities thought to affect the final size [60, 61]. Whilst larger sample sizes are invariably preferred, sample adequacy can also be reflected in any given cohort’s ability to yield richly textured information. Information redundancy and data saturation are also important considerations in this context [62–64].

## Conclusions

The relationship between the built environment and PA is multifaceted and complex. As such, a better understanding of the physical characteristics of neighbourhoods may help us to better understand why a significant proportion of adults do not meet recommended levels of PA. In urban contexts, access to PA facilities, convenient and proximate access to destinations, high residential density, land use, perceived safety and availability of exercise equipment have all been cited as potential determinants of PA levels [8]. As outlined in the COM-B model of change, a particular behaviour will only occur when ‘an individual has the capability and opportunity to engage in the target behaviour and is more motivated to enact that behaviour than any other behaviours’ [65]. Evidence from this exploratory research confirms the availability of a wide variety of high-quality PA infrastructure across the three LGAs. Moreover, there was an overwhelming public appreciation of this infrastructure. As such, we postulate that neither capability nor opportunity, but rather motivation, may be a significant barrier influencing the low habitual PA engagement in these communities. As has been theorised, individual disposition to experience different motivations changes over time and with experience [66]. Therefore, a system-wide approach to promoting PA interventions through environmental, policy and legislative changes to make active choices easier for all, may be required.

## Supplementary Information

**Additional file 1.**

**Additional file 2.**

## Data Availability

The datasets used and/or analysed during the current study are available from the corresponding author on reasonable request.

## Abbreviations

CAPITOL: Critical Age Periods Impacting the Trajectory of Obesogenic Lifestyles
IPAQ-E: International Physical Activity Questionnaire – Environmental module (IPAQ-E)
IS: Incivility score
LGA: Local Government Area
NCD: Non-communicable disease
NW: Northwest
PA: Physical activity
PARA: Physical Activity Resource Assessment
QS: Quality score
SES: Socio-economic status
