# Supplementary material for: Why some do but too many don’t? Barriers and enablers to physical activity in regional Tasmania – an exploratory, mixed-methods study
Source: BMC Public Health. 2022 Mar 31;22:627. doi: 10.1186/s12889-022-13001-6 (PMC8967567; doi:10.1186/s12889-022-13001-6)
Supplement: Supplementary file 1 — Additional file 1. [file 12889_2022_13001_MOESM1_ESM.pdf]

Exploring opportunities for obesity prevention in North-West Tasmania: An evaluation of physical activity infrastructure/ amenities via consumer consultations.

**Your local council (please circle one)**	Burnie	Devonport	Circular Head
**Suburb where you live**			
**Gender** Male Female	Prefer not to say	**Year of birth**	

What is the highest level of education you have completed?

- ☐ Postgraduate Degree
- ☐ Graduate Diploma or Graduate Certificate
- ☐ Bachelor's degree
- ☐ Advanced Diploma or Diploma
- ☐ Certificate III or Certificate IV
- ☐ Secondary education – year 10 or above
- ☐ Certificate I or II
- ☐ Secondary education – year 9 or below

Think about the different facilities in and around your neighbourhood by this we mean the area ALL around your home that you could walk to in **10-15 minutes**.

1. What is the main type of housing in your neighbourhood?

- ☐ Detached single-family housing
- ☐ Townhouses, terrace houses, apartments of 2-3 stories
- ☐ Apartments of 4-12 stories
- ☐ Mix of single-family residences and townhouses, row houses or apartments
- ☐ Don't know/Not sure
- ☐ Home on more than 1 hectare- Rural Living Zone

The next items are statements about your neighbourhood related to walking and bicycling.

2. Many shops, stores, markets or other places to buy things I need are within easy walking distance of my home. Would you say that you…

- ☐ Strongly disagree
- ☐ Somewhat disagree
- ☐ Somewhat agree
- ☐ Strongly agree
- ☐ Don't know/Not sure

3. It is within a 10-15 minutes walk to a transit stop (such as a bus stop) from my home. Would you say that you…

- ☐ Strongly disagree
- ☐ Somewhat disagree
- ☐ Somewhat agree
- ☐ Strongly agree
- ☐ Don't know/Not sure

4. There are footpaths on most of the streets in my neighbourhood. Would you say that you…

- ☐ Strongly disagree
- ☐ Somewhat disagree
- ☐ Somewhat agree
- ☐ Strongly agree
- ☐ Don't know/Not sure

5. There are facilities to bicycle in or near my neighbourhood, such as special lanes, separate paths or trails, shared use paths for cycles and pedestrians. Would you say that you…

- ☐ Strongly disagree
- ☐ Somewhat disagree
- ☐ Somewhat agree
- ☐ Strongly agree
- ☐ Don't know/Not sure

6. My neighbourhood has several free or low-cost recreation facilities, such as parks, walking trails, bike paths, recreation centres, playgrounds, public swimming pools, etc. Would you say that you…

- ☐ Strongly disagree
- ☐ Somewhat disagree
- ☐ Somewhat agree
- ☐ Strongly agree
- ☐ Don’t know/Not sure

7. The crime rate in my neighbourhood makes it unsafe to go on walks at night. Would you say that you…

- ☐ Strongly disagree
- ☐ Somewhat disagree
- ☐ Somewhat agree
- ☐ Strongly agree
- ☐ Don’t know/Not sure

8. There is so much traffic on the streets that it makes it difficult or unpleasant to walk in my neighbourhood. Would you say that you…

- ☐ Strongly disagree
- ☐ Somewhat disagree
- ☐ Somewhat agree
- ☐ Strongly agree
- ☐ Don’t know/Not sure

9. I see many people being physically active in my neighbourhood doing things like walking, jogging, cycling, or playing sports and active games. Would you say that you…

- ☐ Strongly disagree
- ☐ Somewhat disagree
- ☐ Somewhat agree
- ☐ Strongly agree
- ☐ Don’t know/Not sure

10. There are many interesting things to look at while walking in my neighbourhood. Would you say you…

- [ ] Strongly disagree
- [ ] Somewhat disagree
- [ ] Somewhat agree
- [ ] Strongly agree
- [ ] Don't know/Not sure

11. How many motor vehicles in working order (e.g., cars, trucks, motorcycles) are there at your household?

___ motor vehicles

- [ ] Don't know/ not sure

12. There are many four-way intersections in my neighbourhood. Would you say that you…

- [ ] Strongly disagree
- [ ] Somewhat disagree
- [ ] Somewhat agree
- [ ] Strongly agree
- [ ] Don't know/Not sure

13. The footpaths in my neighbourhood are well maintained (paved, with few cracks) and not obstructed. Would you say that you…

- [ ] Strongly disagree
- [ ] Somewhat disagree
- [ ] Somewhat agree
- [ ] Strongly agree
- [ ] Don't know/Not sure

14. Places for bicycling (such as bike paths) in and around my neighbourhood are well maintained and not obstructed. Would you say that you…

- ☐ Strongly disagree
- ☐ Somewhat disagree
- ☐ Somewhat agree
- ☐ Strongly agree
- ☐ Don't know/Not sure

15. There is so much traffic on the streets that it makes it difficult or unpleasant to ride a bicycle in my neighbourhood. Would you say that you…

- ☐ Strongly disagree
- ☐ Somewhat disagree
- ☐ Somewhat agree
- ☐ Strongly agree
- ☐ Don't know/Not sure

16. The crime rate in my neighbourhood makes it unsafe to go on walks during the day. Would you say that you…

- ☐ Strongly disagree
- ☐ Somewhat disagree
- ☐ Somewhat agree
- ☐ Strongly agree
- ☐ Don't know/Not sure

17. There are many places to go within easy walking distance of my home. Would you say that you…

- ☐ Strongly disagree
- ☐ Somewhat disagree
- ☐ Somewhat agree
- ☐ Strongly agree
- ☐ Don't know/Not sure

Please make any comments that apply to the following questions.

Are there any barriers towards you being physically active?

How has COVID19 and the government restrictions affected your physical activity?

Is there anything else you wish to add that you think is relevant?

Thank you for your assistance and your participation!
