# Supplementary material for: Why some do but too many don’t? Barriers and enablers to physical activity in regional Tasmania – an exploratory, mixed-methods study
Source: BMC Public Health. 2022 Mar 31;22:627. doi: 10.1186/s12889-022-13001-6 (PMC8967567; doi:10.1186/s12889-022-13001-6)
Supplement: Supplementary file 2 — Additional file 2. [file 12889_2022_13001_MOESM2_ESM.pdf]

Physical Activity Resource Assessment Instrument (PARA)

1) **Date** ____________ 2) **Data col** _______ 3) **HD/PA Resource ID** ________________

4) **Time**
start:_____
stop:____

5) **Phone Call**
departure:____
arrival:_____

6) **Type of Resource**
1 Outdoor recreation Track
2 Natural amenity and/or green space
3 Sporting venue
4 Multi purpose community centre
5 Gym/Fitness Centre
6 School

7) **Approximate Size: 1** sm **2** med **3** lg

8) **Capacity (indoor)** ______________

9) **Cost**
1 Free
2 Pay at the door
3 Pay for only certain programs
4 Other ______________________

10) **Hours** a) open _______ b) close ______

11) **Signage** – Hours yes ☐ no ☐

12) **Signage** – Rules yes ☐ no ☐

**Feature**	Rating				**Amenity**	Rating			
13) Football Oval	0	1	2	3	26) Access Points	0	1	2	3
14) Basketball courts	0	1	2	3	27) Bathrooms	0	1	2	3
15) Soccer field	0	1	2	3	28) Benches	0	1	2	3
16) Bike Rack	0	1	2	3	29) Drinking fountain	0	1	2	3
17) Exercise Stations	0	1	2	3	30) Fountains	0	1	2	3
18) Play equipment	0	1	2	3	31) Landscaping efforts	0	1	2	3
19) Pool > 1 Metre deep	0	1	2	3	32) Lighting	0	1	2	3
20) Sand Pit	0	1	2	3	33) Picnic tables shaded	0	1	2	3
21) Sidewalk	0	1	2	3	34) Picnic tables no-shade	0	1	2	3
22) Tennis courts	0	1	2	3	35) Shelters	0	1	2	3
23) Trails – running/biking	0	1	2	3	36) Shower/Locker room	0	1	2	3
24) VB courts	0	1	2	3	37) Rubbish bins	0	1	2	3
25) Wading Pool < 1 metre deep	0	1	2	3					

**Incivilities**	Rating				**Incivilities**	Rating			
38) Auditory annoyance	0	1	2	3	44) Graffiti/tagging	0	1	2	3
39) Broken glass	0	1	2	3	45) Litter	0	1	2	3
40) Dog refuse	0	1	2	3	46) No grass	0	1	2	3
41) Dogs Unattended	0	1	2	3	47) Overgrown grass	0	1	2	3
42) Evidence of alcohol use	0	1	2	3	48) Sex paraphernalia	0	1	2	3
43) Evidence of substance use	0	1	2	3	49) Vandalism	0	1	2	3

Comments:
